# Associations between transitions of intrinsic capacity and frailty status, and 3-year disability

**DOI:** 10.1186/s12877-023-03795-4

**Published:** 2023-02-14

**Authors:** Shuli Jia, Wanyu Zhao, Meiling Ge, Xin Xia, Fengjuan Hu, Qiukui Hao, Yan Zhang, Mei Yang, Jirong Yue, Birong Dong

**Affiliations:** 1 National Clinical Research Center of Geriatrics, West China Hospital, Sichuan University, No. 37, Guo Xue Xiang Renmin Nan Lu, Chengdu, 610041 Sichuan China; 2 Center of Gerontology and Geriatrics, West China Hospital, Sichuan University, No. 37, Guo Xue Xiang Renmin Nan Lu, Chengdu, 610041 Sichuan China; 3 School of Rehabilitation Science, McMaster University, Hamilton, ON Canada

**Keywords:** Intrinsic capacity, Frailty, Disability, Healthy aging, Functional decline

## Abstract

**Background:**

The trajectory of frailty and intrinsic capacity (IC) often overlap in older adults. Longitudinal analyses of transitions of frailty and IC, and their associations with incident functional decline are limited. The present study aimed to identify transitions of frailty status and IC, and explore associations between transitions of frailty and IC, and future disability among community-dwelling older adults.

**Methods:**

In the West China and Aging Trend Study, 808 participants aged ≥ 60 years completed baseline and three years follow-up (frailty, IC and disability assessments). Physical frailty was measured based on Fried phenotype. IC was evaluated by five domains (cognition, locomotion, sensory, psychological, and vitality). Disability was defined as a need for assistance in any items in activity of daily living (ADL) or the instrumental activity of daily living (IADL). Logistic regressions were performed to examine their relationships.

**Results:**

Four transitions of IC status (kept well: 27.4%, improved: 8.4%, worsened: 35.4%, and kept poor: 28.8%), and two transitions of frailty status (kept not-frail/improved: 93.2%, kept frail/worsened: 6.8%) were identified. Impaired locomotion and vitality at baseline were significantly associated with kept frail or worsened frail. However, impaired sensory and vitality at baseline not frailty status was significantly associated with transitions of IC. Adjusted for covariates and transitions of frailty, kept poor IC was associated with ADL (OR = 2.26, 95%CI = 1.17,4.34) and IADL disability (OR = 3.74, 95%CI = 1.79, 7.82).

**Conclusions:**

Transitions of IC, but not frailty were associated with higher risk of incident disability. Baseline locomotion and vitality impairment were associated with worsened or kept frail. Our findings support the WHO’s notion of monitoring and optimizing IC to delay deterioration of IC and preventing frailty and disability.

**Clinical trial number:**

ChiCTR1800018895

## Background

The World Health Organization (WHO) introduced the construct of intrinsic capacity (IC) [1] to promote healthy aging, which refers to the composite of all physical and mental capacities of an individual, including five domains: cognition, locomotion, psychological, sensory and vitality [2, 3]. Evidence shows that focusing on IC of older adults could have higher predictability of functional decline compared to the multimorbidity status in older people [4]. In recent years, IC has been increasingly validated and verified in different populations [3, 5]. In the literature, slow gait speed, depression, and hearing loss, which are also among the domains of IC are proven risk factors for adverse health outcomes [6–10]. Charles et al. evaluated the predictive value of IC in Belgian nursing home residents and suggested that the combination of low balance performance and a low mini nutritional assessment (MNA) score predicted the incidence of 3-year mortality and falls [11].

Frailty is a common geriatric syndrome characterized by a decline in physiological systems, which makes an individual more susceptible to stressors and increases the risk of adverse health outcomes [12, 13]. Frailty and IC are concepts both focusing on promoting the development of person-centered care plans and lead to individualized strategies to reverse, delay or prevent age-related functional losses [14]. Currently, the relationship of IC and frailty is still controversial. Robledo et al. argued that the IC score acts as a determinant of frailty, suggesting that the IC indices were significantly associated with frailty [15]. Whilst others argued that IC can be considered as an evolution of the concept of frailty [14]. Monitoring IC can support appropriate evaluation of frailty, and can also help in developing individualized care plans [14]. Liu et al. showed that IC impairment and frailty overlap and co-exist in community older adults, and new impairment in locomotion and vitality are associated with the transitions from non-frail to frail status [16], which suggested that monitoring IC trajectory is crucial for early action to prevent frailty.

However, several questions arise while considering the two entities -frailty and IC in the context of healthy aging, such as which entity should be a priority in older adults? or are they two equally significant tools and can be substituted mutually? Frailty and IC change dynamically with time and are potentially reversible. Longitudinal monitoring of these entities is necessary to better understand the relationship between them. To the best of our knowledge, few studies have investigated the longitudinal transitions of frailty and IC together and explored the associations between them. Therefore, the major aim of this study was to investigate three-year transitions of IC and frailty and their associations among community-dwelling older people based on a longitudinal study. Additionally, previous evidence has confirmed that IC impairment and frailty are associated with disability and other adverse events [3, 11, 17]. However, how the transitions of IC and frailty predict physical disability remains unknown. So, the secondary aim of our study was to explore the associations between transitions of IC and frailty, and incident disability, which may facilitate to untangle the relationship of frailty and IC.

## Methods

### Study design and population

**Fig.1:**
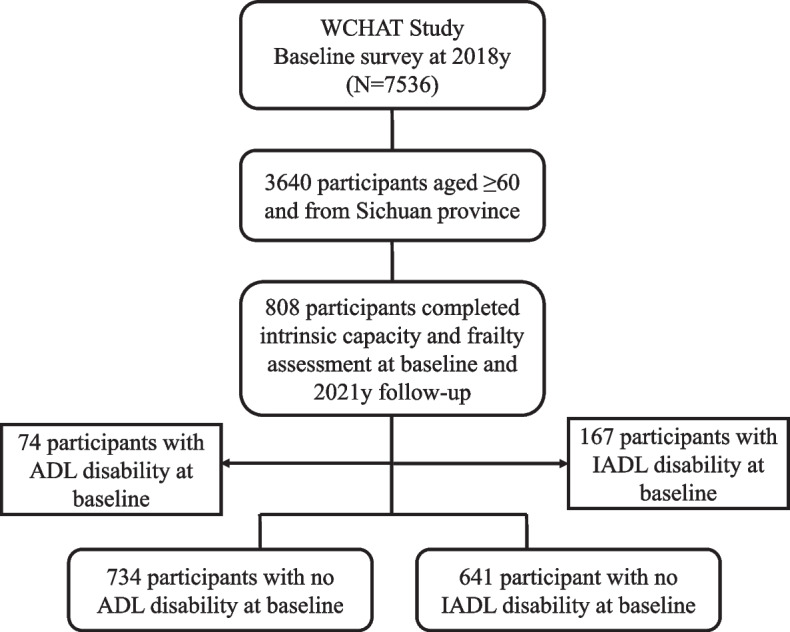
Study flow. ADL, activity of daily living; IADL, instrumental activity of daily living

Data used in this study were from the West China Health and Aging Trend (WCHAT) study, an ongoing prospective cohort study composed of multi-ethnic community-dwelling adults (≥ 50 year) in west China. Details of the methodology and study design of this cohort have been published elsewhere [18]. In brief, a baseline survey was conducted in July 2018, involving 7,536 community-dwelling individuals aged ≥ 50 years. Face-to-face interviews and a battery of physical examinations were completed during the baseline visit, and participants were invited for follow-up assessments every year by face-to-face interview or phone. In this study, data collected at baseline and 3-year follow-up visit (July 2021) was considered (Fig. 1). In this study, we focused on individuals aged ≥ 60 years from Sichuan province (*N* = 3640). A total of 808 participants with complete data on IC and frailty at baseline and follow-up were included. This study was registered in the Chinese Clinical Trial Registry (ChiCTR1800018895). All study participants provided informed consent. Legally authorized representatives of illiterate participants provided informed consent for the study. The Ethics Committee of West China Hospital Sichuan University approved this study (reference: 2017–445).

### Definition of IC impairment

IC impairment (ICI) was defined by assessing the five domains of IC: cognition, psychosocial, sensory, vitality and locomotion. Participants meeting 2 or more impaired domains were categorized as ≥ 2 ICI, those with only one domain impairment were considered to be 1 ICI, and those with none were categorized as 0 ICI.

Cognition was evaluated by a 10-item Short Portable Mental Status Questionnaire (SPMSQ). Participants scored ≥ 3 were regarded as cognition impairment [19].
2)Psychological domain was measured with the 15-item Geriatric Depression Scale (GDS-15) [20]. The scores ≥ 5 represents psychological impairment.
Sensory function was assessed using two self-reported questions for vision and hearing impairment: (1) Can you see clearly with or without glasses? Participants were considered as having a vision impairment if the answer was nearly invisible or totally blind. (2) Can you clearly hear others in daily life? Participants were considered as having a hearing impairment if the answers were- often need others to raise voice or slow down speaking rate to clearly hear and almost deaf or completely deaf. Impairment of either vision or hearing was considered as having a sensory impairment.
Vitality was evaluated with the Mini Nutrition Assessment-Short form (MNA-SF) [21]. The risk of malnutrition (the sum score < 12) and malnutrition (the sum score < 8) were regarded as vitality impairment.
Locomotion was assessed by the Short Physical Performance Battery test (SPPB) [22]. The sum score ≤ 9 was deemed as locomotion impairment.
IC was evaluated at baseline (2018) and three years follow-up (2021). We defined transitions of IC as four categories: 1) kept well: ≤ 1 ICI at both baseline and 3-year follow-up survey; 2) improved: ≥ 2 ICI at baseline and ≤ 1 ICI at 3-year follow-up; 3) Worsened: ≤ 1 ICI at baseline, and ≥ 2 ICI at 3-year follow-up; 4) kept poor: ≥ 2 ICI domains at both baseline and 3-year follow-up.

### Frailty assessment

Frailty was assessed by the modified physical frailty phenotype [23], which included 5 physical phenotype components: shrinking, slowness, weakness, exhaustion, and.

low physical activity. Participants who met ≥ 3 components were categorized as frail or having frailty, 1–2 components as pre-frail, and 0 as robust [23]. Shrinking was defined by self-reported weight loss of ≥ 4.5 kg over the last year or BMI < 18.5 kg/m2 [24]. Slowness was measured by the time to walk 4 m and defined as being ≤ 20^th^ percentile of sex and height adjusted time. Weakness was evaluated using the maximal handgrip strength of the dominant hand of 2 trials (EH101; Camry, 130 Zhongshan, China) and defined as being ≤ 20^th^ percentile of sex and BMI adjusted value (low weight: BMI < 18.5 kg/m2; normal: BMI 18.5–23.9 kg/m2; overweight: BMI 24–27.9 kg/m2; and obese: BMI ≥ 28 kg/m2). Exhaustion was defined as meeting either of the following 3 criteria: (1)feeling excessively fatigue for most of the time; (2)feeling excessively weak for most of the time; or (3) self-rated energy level was ≤ 3 (in a scale of 1–10, when 10 represents the highest energy level and 1 the lowest) [25]. Low physical activity was defined by being ≤ the 20th percentile of energy consumption per week stratified by gender. Energy consumption was measured by a validated China Leisure Time Physical Activity Questionnaire (CLTPAQ) [26, 27], which was a modified version of the Minnesota Leisure Time Physical Activity Questionnaire (MLTPAQ) [28] according to the Chinese lifestyle and cultural background.

We defined transition of frailty as two status: 1) kept not-frail/improved (not-frail in 2018 and 2021, or frail in 2018 and not-frail in 2021; 2) kept frail/worsened: frail in 2018 and 2021, or not-frail in 2018 and frail 2021.

### Three-year disability

Disability was assessed by activity of daily living (ADL) and instrumental activity of daily living (IADL) disability, which were assessed in 2018 and 2021. ADL disability was defined as a need for assistance in any items in Barthel Index [29]. IADL disability was defined as a need for assistance in any items in IADL [30].

### Covariates

Demographics (age, sex), ethnicity (Han and others), education levels (illiterate, elementary school, middle school, and high school and above), marital status (married, unmarried/widowed/divorced), lifestyles (smoking and drinking), and number of self-reported history of diagnosed chronic diseases (hypertension, heart diseases, chronic obstructive pulmonary diseases, liver and renal diseases, gastrointestinal diseases, diabetes, stroke, arthritis, and cancer) were obtained at baseline survey.

### Statistical methods

Data were expressed as the mean and standard deviation for continuous data. Categorical data were expressed as numbers and percentages. Group differences were evaluated using the ANOVA test for continuous variables and the chi-square or Fisher’s exact tests for categorical variables. The association between transitions of IC and frailty, and disability were examined by logistic regression analyses. Models were adjusted for age, sex, ethnicity, education, marital status, smoking and drinking, multimorbidity, baseline frailty status, and IC impairment. We obtained odds ratios (ORs) for transitions of IC and frailty and 95% confidence intervals (CIs) between the groups. Stata 15.1 (Stata Corp, College Station, TX, USA) was used for statistical analyses. Two-sided *p* < 0.05 was considered statistically significant.

## Results

### Characteristics of baseline study population

Table 1 shows baseline characteristics of the 808 participants. Among these, mean age was 67.8 years, 59.5% were female, 53.3% were of Han ethnicity, 81.1% were married, 34.4% were illiterate, and 15.8% reported ≥ 2 chronic diseases. The baseline prevalence of frailty was 2.6%. Considering individual IC domains, sensory and locomotion impairment were the two most common IC impairment domains (44.6% and 36.5%, respectively). At baseline, only 24.9% had intact IC. Participants with more domains of IC impairment were older, had lower education, and of higher prevalence of frailty, IADL and ADL disability at baseline (Table 1).

**Table 1:** Baseline characteristics of the study participants according to the baseline number of IC impaired domains. (*N* = 808)

Variables	Total	0 ICI	1 ICI	≥ 2 ICI	*P* value
Number of participants (%)	808	201(24.9)	306(37.9)	301(37.2)	
Age(years), mean (SD)	67.8(5.1)	66.0(3.8)	67.8(5.0)	69.0(5.7)	< 0.001
Female, n (%)	481(59.5)	116(57.7)	179(58.5)	186(61.9)	0.59
Han ethnicity, n (%)	431(53.3)	98(48.8)	170(55.6)	163(54.2)	< 0.30
Marital status, n (%)					< 0.51
Married	655(81.1)	164(81.6)	253(82.7)	238(79.1)	
Divorced/widowed/single	153(18.9)	37(18.4)	53(17.3)	63(20.9)	
Education, n (%)					< 0.001
Illiterate	278(34.4)	49(24.4)	100(32.7)	129(42.9)	
Elementary school	378(46.8)	101(50.2)	147(48.0)	130(43.2)	
Middle school	132(16.3)	47(23.4)	51(16.7)	34(11.3)	
High school or higher	20(2.5)	4(2.0)	8(2.6)	8(2.7)	
Smoking history, n (%)	148(18.3)	38(19.0)	55(18.0)	55(18.3)	0.96
Drinking, n (%)	240(29.7)	73(36.3)	84(27.5)	83(27.6)	0.061
Number of Chronic diseases, n (%)					0.39
0	480(59.4)	127(63.2)	186(60.8)	167(55.5)	
1	200(24.8)	47(23.4)	75(24.5)	78(25.9)	
≥ 2	128(15.8)	27(13.4)	45(14.7)	56(18.6)	
Frailty, n (%)					< 0.001
Robust	434(53.7)	145(72.1)	174(56.9)	115(38.2)	
Pre-frail	353(43.7)	56(27.9)	127(41.5)	170(56.5)	
Frail	21(2.6)	0(0)	5(1.6)	16(5.3)	
ADL disability, n (%)	74(9.2)	11(5.5)	23(7.5))	40(13.3)	0.005
IADL disability, n (%)	167(20.7)	25(12.4)	61(19.9)	81(26.9)	< 0.001
Impaired cognition, n (%)	92(11.4)	/	19(6.2)	73(24.3)	< 0.001
Impaired locomotion, n (%)	295(36.5)	/	97(31.7)	198(65.8)	< 0.001
Impaired sensory, n (%)	360(44.6)	/	133(43.5)	227(75.4)	< 0.001
Impaired psychological, n(%)	137(17.0)	/	32(10.5)	105(34.9)	< 0.001
Impaired vitality, n (%)	157(19.4)	/	25(8.2)	132(43.9)	< 0.001

*IC* Intrinsic capacity, *ICI* Intrinsic capacity impairment, *ADL* Activity of daily living, *IADL* Instrumental activity of daily living

### Three-year transitions of IC and frailty

**Fig. 2:**
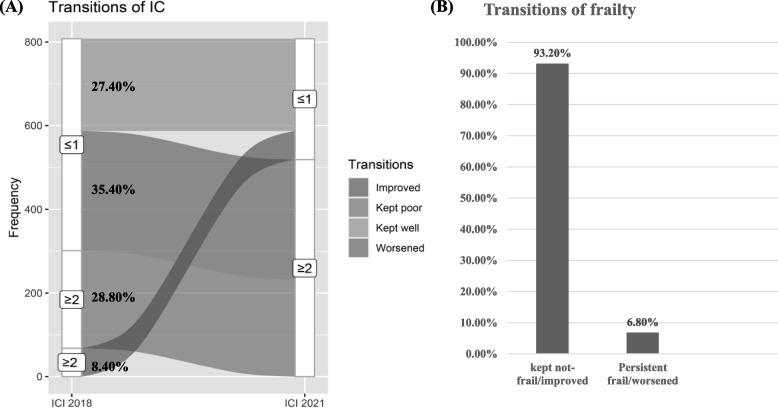
Transitions of IC and frailty over three years period. **A** Transitions of intrinsic capacity, IC, intrinsic capacity; ICI, intrinsic capacity impairment; **B** Prevalence of frailty transitions

The three-year transitions of IC and frailty are presented in Fig. 2. Through three years period, 93.2% participants kept not-frail or improved (transitions from frail to not-frail), and 6.8% kept frail or worsened (transitions from not-frail to frail) (Fig. 2B). For IC, only 8.4% individuals had improved IC (from ≥ 2 ICI to 1 ICI), and 27.4% had a relatively well IC, 28.8% kept poor, and 35.4% had worsened IC (Fig. 2A).

In order to identify associations between baseline IC and frailty status and 3-year transitions of IC and frailty, logistic regressions were performed (Table 2). Baseline prefrail and frail were significantly associated with kept frail and worsened frail. Baseline locomotion (OR = 1.99, 95%CI:1.04,3.80) and vitality (OR = 2.10, 95%CI: 1.10,4.00) impairment were significantly associated with kept frail or worsened frail status. Baseline frailty status was not significantly associated with transitions of IC. Baseline sensory and vitality impairment were associated with worsened IC and kept poor IC.

**Table 2:** Associations of baseline IC and frailty status and transitions of IC and frailty

Baseline frailty and IC	Kept frail/worsened vs kept not-frail/improved	Kept poor/worsened vs kept well/improved IC
	**Adjusted **^**a**^** OR 95%CI**	**Adjusted **^**a**^** OR 95%CI**
Frailty		
Robust	Reference (1)	Reference (1)
Prefrail	2.60(1.27, 5.29)**	1.26(0.91,1.75)
Frail	4.13(1.04,16.41)*	0.72(0.24,2.10)
Impaired cognition	1.38(0 .62,3.06)	1.18(0.67,2.08)
Impaired locomotion	1.99(1.04,3.80)*	1.36(0.96,1.93)
Impaired psychological	1.20(0 .57,2.50)	1.36(0.88,2.09)
Impaired sensory	1.00(0 .53,1.85)	1.60(1.16,2.21)**
Impaired vitality	2.10(1.10,4.00)*	2.18(1.39,3.43)**

*IC* Intrinsic capacity, *OR* Odds ratio, *CI* Confident interval

^a^ adjusted age, sex, ethnicity, education, marital, smoking, drinking, number of chronic diseases, baseline frailty status, and baseline impaired IC domains

^*^p < .05 **p < .01 ***p < 0.001

### Associations between transitions of IC and frailty and 3-year disability

Table 3 shows associations between transitions of IC and frailty and disability in IADL and ADL in 2021(excluded participants who had ADL disability or IADL disability at baseline). Kept poor IC (vs kept well IC) was associated with higher risk of incident ADL and IADL disability when controlling for covariates and transitions of frailty (OR = 2.26, 95%CI = 1.17,4.34 and OR = 3.74, 95%CI = 1.79,7.82, respectively). Transitions of frailty was not significantly associated with new incidence of ADL and IADL disability when adjusting for confounders and transitions of IC.

**Table 3:** Association between transitions of IC and frailty and 3-year ADL and IADL disability

IC and frailty	ADL disability ^a^		IADL disability ^b^	
	**Multivariable model OR (95% CI)**	***P* value**	**Multivariable model** **OR (95% CI)**	***P* value**
**Transitions of IC**				
Kept well	1(reference)	/	1(reference)	/
Improved	1.61(0.65–3.99)	0.307	0.73(0.19–2.79)	0.648
Worsened	1.70(0.89–3.23)	0.108	1.46(0.68–3.14)	0.331
Kept poor	2.26(1.17–4.34)	0.015	3.74(1.79–7.82)	< 0.001
**Transitions of frailty**				
Kept not-frail or improved	1(reference)	/	1(reference)	/
Kept frail or worsened	1.72(0.81–3.65)	0.196	1.87(0.79–4.41)	0.155

*IC* Intrinsic capacity, *ADL* Activity of daily living, *IADL* Instrumental activity of daily living, *OR* Odds ratio, *CI* Confident interval

^**a**^ *N* = 734 adjusted age, sex, ethnicity, education, marital status, smoking, drinking, number of chronic diseases, transitions of frailty and IC

^b^ *N* = 641 adjusted age, sex, ethnicity, education, marital status, smoking, drinking, number of chronic diseases, transitions of frailty and IC

## Discussion

This study described the profile of transitions of IC and frailty among Chinese older adults in Sichuan province, and explored the relationships of transitions of IC and frailty, and incidence of disability. Our key findings were mostly in line with the literature. Baseline frailty status, locomotion and vitality impairment were significantly associated with transitions of frailty, even after adjusting for IC impairment. Transitions of IC but not frailty was found to predict future disability at three years..

Our study showed IC impairment was common among community-dwelling older adults. Nearly one-third of samples experienced worsened IC during three years period. Sensory (44.6%) and locomotion (36.5%) were the two most prevalent impaired IC domains, which is consistent with the findings from a large sample of population-based study conducted by Ma et al. in China [31].

We explored the effect of baseline IC and frailty status on the transitions of IC. Baseline sensory and vitality impairment were independently associated with worsened or persistent poor IC, which indicated that improving any of the two domains of IC may delay or prevent deterioration of IC. Sensory impairment was the most prevalent IC impairment in older adults. Sensory impairment can lead to subsequent cognitive decline [32], depression [33], and decreased mobility. However, baseline frailty status was not associated with IC transitions in our study. We also analyzed the effect of baseline IC and frailty status on the transitions of frailty. Baseline locomotion and vitality impairment was significantly associated with frailty transitions, which was consistent with a previous study conducted in China [16]. The authors found that new impairment in locomotion and vitality to be associated with the transitions from non-frail to frail status [16], which may be explained by the definition of frailty per se. Shrinking and slowness, the two components of physical frailty, may be the results of poor vitality and locomotory impairment. These findings support the concept of frailty being an adverse outcome of IC deterioration during the process of aging [15]. Therefore, screening for IC may be essential to prevent frailty. Interventions to optimize locomotory capacity and vitality such as individualized exercise training and nutritional supplementation have been shown to reverse frailty status, which may also have important implications for optimization of IC.

Previous study showed IC impairment was significantly associated with IADL and ADL disability [3]. Impairment in an additional IC domain demonstrated a higher risk of incident IADL and ADL disability by 27% and 23% over 5 years, respectively [34]. Subjects with a higher impairment in IC domains at baseline showed higher odds of ADL disability (OR = 9.51 for impairment in ≥ 3 domains) [4]. Our study also demonstrated that IC transitions can predict IADL and ADL disability, which is in line with a previous study conducted by Stolz et al. [35]. The authors found that declined IC value was associated with increased risk of ADL disability, nursing home stay, and mortality. However, frailty transitions were not significantly associated with ADL or IADL disability in the present study. We can explain this result from following perspectives. Compared to IC impairment, the prevalence of frailty was relatively low, and frailty status was stable during three years. In this study, most participants kept not-frail, and only a small number of participants were persistent frail or transitions from robust to frail. So, there may be potential bias. Further studies are needed to confirm our findings in other larger cohorts. Therefore, dynamic monitoring of IC could work as an early warning system for informing prevention and intervention of frailty.

Our study has several strengths. We not only identified the three-year transitions of IC and frailty, but also investigated the relationship between them and disability, which helps us understand the relationship of frailty and IC, and also provides some information on the improvement of IC and prevention of frailty and functional decline. However, certain limitations should be noted. First, the sample of the present study was relatively small. The events of participants with kept frailty or worsened frailty were not enough, which might have weakened the statistical power and the reliability of conclusion. Additionally, the data collected were from the Western region in China, so our study population might not be representative of the general community-dwelling older population in China. Finally, IC measurements in our study differed with other studies, nevertheless, there is no gold standard for assessing IC yet. A validated and standardized method of IC assessment is needed to maintain consistency between studies.

## Conclusions and implications

In conclusion, transitions of IC had predictive value in incidence of ADL and IADL disability. Baseline locomotion and vitality impairment were associated with transitions from not-frail to frail or persistent frail status and IC decline. Therefore, monitoring IC trajectory should be a priority to develop individualized interventions for optimization of IC and preventing frailty and disability. Additionally, clinicians should be aware of the increased risk of IC decline and frailty for older adults, in particular with locomotion impairment and malnutrition.

## Data Availability

The datasets used and/or analyzed during the current study available from the corresponding author on reasonable request.

## Abbreviations

ADL: Activities of daily living
ANOVA: Analysis of Variance
BMI: Body mass index
CLTPAQ: China Leisure Time Physical Activity Questionnaire
CI: Confidence interval
GDS-15: 15-Iterm Geriatric Depression Scale
IC: Intrinsic capacity
IADL: Instrumental activity of daily living
ICI: Intrinsic capacity impairment
MLTPAQ: Minnesota Leisure Time Physical Activity Questionnaire
MNA: Mini nutritional assessment
MNA-SF: Mini Nutrition Assessment-Short form
OR: Odds ratio
SD: Standard deviations
SPPB: Short Physical Performance Battery
SPMSQ: Short Portable Mental Status Questionnaire
WCHAT: West China Health and Aging Trend
